# Exonic sequencing identifies *TLR1* genetic variation associated with mortality in Thais with melioidosis

**DOI:** 10.1080/22221751.2019.1575172

**Published:** 2019-02-19

**Authors:** Shelton W. Wright, Mary J. Emond, Lara Lovelace-Macon, Deirdre Ducken, James Kashima, Viriya Hantrakun, Wirongrong Chierakul, Prapit Teparrukkul, Narisara Chantratita, Direk Limmathurotsakul, T. Eoin West

**Affiliations:** a Division of Pediatric Critical Care Medicine, Department of Pediatrics, University of Washington, Seattle, WA, USA; b Department of Biostatistics, University of Washington, Seattle, WA, USA; c Division of Pulmonary, Critical Care and Sleep Medicine, Department of Medicine, University of Washington, Seattle, WA, USA; d Mahidol-Oxford Tropical Medicine Research Unit, Faculty of Tropical Medicine, Mahidol University, Bangkok, Thailand; e Department of Clinical Tropical Medicine, Faculty of Tropical Medicine, Mahidol University, Bangkok, Thailand; f Department of Internal Medicine, Sunpasitthiprasong Hospital, Ubon Ratchathani, Thailand; g Department of Microbiology and Immunology, Faculty of Tropical Medicine, Mahidol University, Bangkok, Thailand; h Department of Tropical Hygiene, Faculty of Tropical Medicine, Mahidol University, Bangkok, Thailand; i International Respiratory and Severe Illness Center, University of Washington, Seattle, WA, USA

**Keywords:** TLR1, melioidosis, sepsis, genetic variation, *Burkholderia pseudomallei*

## Abstract

Melioidosis, an infectious disease caused by the bacterium *Burkholderia pseudomallei*, is a common cause of sepsis in Southeast Asia. We investigated whether novel *TLR1* coding variants are associated with outcome in Thai patients with melioidosis. We performed exonic sequencing on a discovery set of patients with extreme phenotypes (mild vs. severe) of bacteremic melioidosis. We analysed the association of missense variants in *TLR1* with severe melioidosis in a by-gene analysis. We then genotyped key variants and tested the association with death in two additional sets of melioidosis patients. Using a by-gene analysis, *TLR1* was associated with severe bacteremic melioidosis (*P* = 0.016). One of the eight *TLR1* variants identified, rs76600635, a common variant in East Asians, was associated with in-hospital mortality in a replication set of melioidosis patients (adjusted odds ratio 1.71, 95% CI 1.01–2.88, *P* = 0.04.) In a validation set of patients, the point estimate of effect of the association of rs76600635 with 28-day mortality was similar but not statistically significant (adjusted odds ratio 1.81, 95% CI 0.96–3.44, *P* = 0.07). Restricting the validation set analysis to patients recruited in a comparable fashion to the discovery and replication sets, rs76600635 was significantly associated with 28-day mortality (adjusted odds ratio 3.88, 95% CI 1.43–10.56, *P* = 0.01). Exonic sequencing identifies *TLR1* as a gene associated with a severe phenotype of bacteremic melioidosis. The *TLR1* variant rs76600635, common in East Asian populations, may be associated with poor outcomes from melioidosis. This variant has not been previously associated with outcomes in sepsis and requires further study.

## Introduction

*Burkholderia pseudomallei,* a Gram-negative, flagellated bacillus, causes melioidosis and is characterized as a Tier 1 bioterrorism agent by the US Centers for Disease Control and Prevention. Melioidosis is endemic to southeast Asia as well as northern Australia, and while its worldwide prevalence is poorly characterized, it may well be a global threat [1–3]. Recent modelling suggests that roughly 165,000 cases occur worldwide with an estimated 54% mortality rate [4]. Specific sub-populations, including those with diabetes, are at particularly increased risk of melioidosis [5]. The clinical spectrum of infection ranges from asymptomatic seropositivity to acute sepsis to more chronic disease [6]. In northeast Thailand, where *B. pseudomallei* is the second most common Gram-negative etiology of bacteraemia, mortality from melioidosis exceeds 40% despite appropriate antibiotic treatment [2].

Given the burden of melioidosis, understanding the mechanisms of the host immune response to infection are critical for future vaccine and therapeutic development. Melioidosis is often characterized by a robust inflammatory host response [7–10]. The activation of Toll-like receptors (TLRs), components of the host innate immune response, during *B. pseudomallei* infection is also well described [11,12]. TLR4, sensing endotoxin (lipopolysaccharide) during Gram-negative infection, and TLR5, sensing bacterial flagellin, play crucial roles in the host inflammatory cascade in response to *B. pseudomallei* [13–15]. Genetic variants in both *TLR4* and *TLR5* are associated with susceptibility to and mortality in melioidosis, respectively [16,17].

TLRs1 and 2 form heterodimers that interact with lipopeptides, peptidoglycan and other bacterial cell wall elements [18]. *B. pseudomallei* can stimulate prompt activation of the innate immune response after recognition by TLR1/2 [11]. Globally, polymorphisms in *TLR1* are associated with outcomes in malaria and *Helicobacter pylori* infection [19–22]. In North American patients, *TLR1* variants are associated with outcomes in sepsis, including survival [23,24]. However, when we analysed previously described *TLR1* variants in Thai subjects with melioidosis, we found no association with organ failure or mortality [25]. Importantly, the frequencies of these variants differ in white American and European populations compared to Southeast Asian populations. The global diversity in *TLR1* genetic architecture may play a role in the response to sepsis.

We hypothesized that novel *TLR1* coding variants exist in Thai subjects and that these variants may be associated with mortality in melioidosis. We *a priori* identified *TLR1* as a candidate gene for investigation in a whole exome sequencing study of patients with extreme phenotypes of bacteremic melioidosis. Extreme phenotype designs increase power to detect associations of rare functional genetic variants [26–28]. We subsequently validated our findings in additional sets of patients with melioidosis.

## Results

### TLR1 variants are associated with extreme phenotypes of bacteremic melioidosis

In order to identify novel *TLR1* variants associated with severe melioidosis, we performed whole exome sequencing of a discovery set of 87 Thai adults with extreme phenotypes of bacteremic melioidosis. The demographics and risk factors of this set are shown in Table 1. Fifty-two percent (45/87) of patients in the discovery set were categorized as the mild phenotype (i.e. subjects surviving to discharge with no organ failure). Patients in the mild phenotype group tended to be younger (49 years vs. 52.5 years, *P* = 0.09) and more frequently female (47% vs. 29%, *P* = 0.08) than those in the severe phenotype group (i.e. subjects with organ failure dying within five days of admission). Both groups had similar rates of diabetes mellitus, chronic liver disease and chronic kidney disease.

**Table 1.** Patient characteristics by cohort set.

	Discovery group				Replication group				Validation group			
	All (*n* = 87)	Mild phenotype (*n* = 45)	Severe phenotype (*n* = 42)	*P* value	All (*n* = 459)	Survivors (*n* = 374)	Non-survivors (*n* = 85)	*P* value	All (*n* = 189)	Survivors (*n* = 90)	Non-survivors (*n* = 99)	*P* value
Baseline characteristics												
Age (median with IQR)	51 (40–60)	49 (39–58)	53 (44–62)	0.09	50 (40–60)	49 (39–60)	55 (42–62)	0.02	54 (46–64)	55 (45–67)	54 (46–64)	0.62
Sex-Male (%)	33 (38)	24 (53)	30 (71)	0.08	243 (53)	199 (53)	44 (52)	0.81	130 (69)	67 (74)	63 (64)	0.12
Sex-Female (%)	54 (62)	21 (47)	12 (29)	0.08	216 (47)	175 (47)	41 (48)	0.81	59 (31)	23 (26)	36 (36)	0.12
Pre-existing conditions												
Diabetes (%)	63 (75)	35 (80)	28 (70)	0.31	228 (50)	194 (53)	34 (41)	0.05	88 (47)	43 (48)	45 (45)	0.75
Chronic liver disease (%)	3 (4)	1 (2)	2 (5)	0.61	6 (1)	5 (1)	1 (1)	1.0	7 (4)	4 (4)	3 (3)	0.71
Chronic kidney disease (%)	5 (6)	2 (5)	3 (7)	0.67	32 (7)	18 (5)	14 (16)	<0.001	27 (14)	7 (8)	20 (20)	0.02
Bactaeremia	87 (100)	45 (100)	42 (100)		188 (41)	134 (36)	54 (64)	<0.001	150 (79)	59 (65)	91 (92)	<0.001

**Figure 1.**
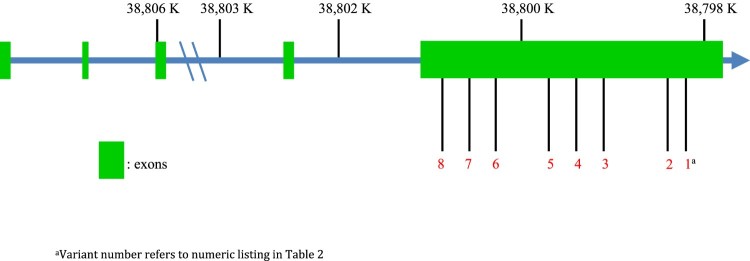
*TLR1* variants of extreme phenotypes of melioidosis in the discovery set. Locations of eight variants in *TLR1* identified during exonic sequencing of extreme phenotypes of Thai patients with melioidosis. Variant numeration is according to Table 2.

Exonic sequencing identified fourteen variants in *TLR1*, of which eight were missense variants and therefore considered for analysis (Table 2). Predicted variant effects were estimated using the Ensembl online Variant Effect Prediction tool [29]. Population-specific minor allele frequencies (MAF) for identified variants were reported from the 1,000 Genomes Project and also calculated within the cohort [30]. All identified variants were located within a single exon of *TLR1* (Figure 1). In a by-gene analysis, utilising a variance components test (adj-SKAT-O), *TLR1* was associated with the severe phenotype of melioidosis (*P* = 0.016).

**Table 2.** Characteristics of missense *TLR1* variants identified by exome sequencing of discovery set subjects.

Variant location^a^	rs#^b^ dbSNP	Allele^c^	Amino acid^d^	Variant Effect Predictor (VEP) impact^e^	Population minor allele frequency (MAF)^f^			Cohort set MAF
					AFR	EUR	EAS	
1. c4p38798703	rs771205452	G/A	Val/Ile	Moderate	<0.01	<0.01	<0.01	0.01
2. c4p38798855	–	C/T	Gly/Glu	Moderate				0.01
3. c4p38799444	rs200457447	C/A	Arg/Cys	Moderate	<0.01	<0.01	<0.01	0.01
4. c4p38799572	–	T/C	Tyr/Cys	Moderate				0.01
5. c4p38799710	rs4833095	A/G	Asn/Ser	Moderate	0.12	0.72	0.40	0.48
6. c4p38800101	rs5743612	C/G	His/Tyr	Moderate	0.12	<0.01	0.01	0.01
7. c4p38800323	rs76600635	T/C	Ser/Pro	Moderate	<0.01	<0.01	0.1	0.18
8. c4p38800412	–	A/G	Ile/Thr	Moderate				0.01

^a^Variant location refers to GRCh37/hg19 location: chromosome (c) and position (*p*).

^b^rs refers to reference SNP cluster ID, when previously identified.

^c^Reference allele listed first, variant allele listed subsequently.

^d^Reference amino acid listed first, variant amino acid listed subsequently.

^e^VEP impact refers to the Ensembl classification of variant sequences, in agreement with the open source tool SNPEff. Moderate impact refers to: “A non-disruptive variant that might change protein effectiveness.” (https://uswest.ensembl.org/Multi/Help/Glossary. Accessed 20 Sept 2018).

^f^Minor allele frequency for each known variant in sub-Saharan African (AFR), European (EUR), and East Asian (EAS) populations per 1,000 Genomes Project. (http://phase3browser.1000genomes.org/index.html. Accessed 20 Sept 2018).

### TLR1 variant rs76600635 is associated with in-hospital mortality in a replication set of melioidosis patients

In order to replicate these findings, we genotyped the eight identified *TLR1* variants in a replication set of 459 Thai adults with melioidosis. Demographic and risk factor data are shown in Table 1. Nineteen percent (85/459) of patients died during hospitalization. Survivors to discharge were significantly younger than non-survivors (*P* = 0.02), more likely to have a prior diagnosis of diabetes (*P* = 0.05) and less likely to have chronic kidney disease (*P* < 0.001). Furthermore, 36% (134/374) of survivors were bacteremic compared to 64% (54/85) of non-survivors (*P* < 0.001).

Five *TLR1* sites demonstrated no or minimal variation (MAF < 0.005) in the replication set. The remaining three *TLR1* variants: rs4833095, rs5743612 and rs76600635, had MAFs of 0.49, 0.03 and 0.15 respectively. These three variants were analysed for an association with hospital mortality (Table 3). We first confirmed the lack of deviation from Hardy–Weinberg equilibrium in survivors (rs4833095 *P* = 0.92; rs5743612 *P* = 0.29, rs76600635 *P* = 0.30). In an unadjusted logistic regression analysis, assuming a dominant model of inheritance, there was no association with death for either rs4833095 or rs5743612 [odds ratio (OR) 1.0, 95% confidence interval (CI) 0.59–1.71, *P* = 0.99; OR 0.59, 95% CI 0.17–2.0, *P* = 0.39, respectively]. In a multivariable dominant model adjusting for age, sex, prior history of diabetes and chronic kidney disease, the lack of association of rs4833095 and rs5743612 with death remained unchanged [adjusted OR 0.97, 95% CI 0.55–1.69, *P* = 0.9 and adjusted OR 0.56, 95% CI 0.15–2.07, *P* = 0.39, respectively]. In contrast, the rs76600635 variant was significantly associated with death in both an unadjusted model [OR 1.72, 95% CI 1.05–2.83, *P* = 0.03] and an adjusted model [adjusted OR 1.71, 95% CI 1.01–2.88, *P* = 0.04].

**Table 3.** Association of *TLR1* variants with hospital mortality in replication set subjects.

Variant	Genotype	Hospital mortality		Unadjusted			Adjusted^a^		
		Survivor (*n* = 374)	Non-survivor (*n* = 85)	OR	95% CI	*P* value	OR	95% CI	*P* value
rs4833095	CC	97 (26%)	22 (26%)						
	CT	189 (50%)	45 (53%)	1.0	0.59–1.71	0.99	0.97	0.55–1.69	0.9
	TT	88 (24%)	18 (21%)						
rs5743612	CC	352 (94%)	82 (96%)						
	CT	21 (6%)	3 (4%)	0.59	0.17–2.0	0.39	0.56	0.15–2.07	0.39
	TT	1 (<1%)	0						
rs76600635	AA	277 (74%)	53 (62%)						
	AG	87 (23%)	30 (35%)	1.72	1.05–2.83	0.03	1.71	1.01–2.88	0.04
	GG	10 (3%)	2 (3%)						

^a^For adjusted model, *P* values were determined using logistic regression analyses adjusted for age, sex, prior history of diabetes mellitus and chronic renal insufficiency.

### TLR1 variant rs76600635 is associated with 28-day mortality and worse survival in a validation set of melioidosis patients

We next sought to validate the association of rs76600635 with mortality in a third group of melioidosis patients. Demographic and risk factor data for this validation set are shown in Table 1. We genotyped rs76600635 in 189 Thai adults who met sepsis criteria, were enrolled within 24 h of admission to the study hospital, and were subsequently diagnosed with melioidosis. In this validation set, 52% (99/189) of patients with melioidosis died within 28 days of hospital admission. Non-survivors were significantly more likely to have pre-existing chronic kidney disease (*P* = 0.02) and bacteraemia (*P* < 0.001).

The MAF of rs76600635 was 0.17. We determined the lack of deviation from Hardy–Weinberg equilibrium in survivors (*P* = 0.65). In a dominant model, the point estimate of effect for the association of rs76600635 with death was similar to the replication set although the association did not quite meet the threshold for significance [unadjusted OR 1.84, CI 0.98–3.47, *P* = 0.06] (Table 4). In a multivariate dominant model adjusting for age, sex, and chronic kidney disease, the association changed minimally [adjusted OR 1.81, 95% CI 0.96–3.44, *P* = 0.07].

**Table 4.** Association of *TLR1* variant rs76600635 with 28-day mortality in validation set subjects.

Variant	Genotype	28-day mortality		Unadjusted			Adjusted^a^		
		Survivor (*n* = 90)	Non-survivor (*n* = 99)	OR	95% CI	*P* value	OR	95% CI	*P* value
rs76600635	AA	68 (76%)	62 (63%)						
	AG	20 (22%)	35 (35%)	1.84	0.98–3.47	0.06	1.81	0.96–3.44	0.07
	GG	2 (2%)	2 (2%)						

^a^ For adjusted model, values were determined using logistic regression analyses adjusted for age, sex, prior history of diabetes mellitus and chronic renal insufficiency.

**Figure 2.**
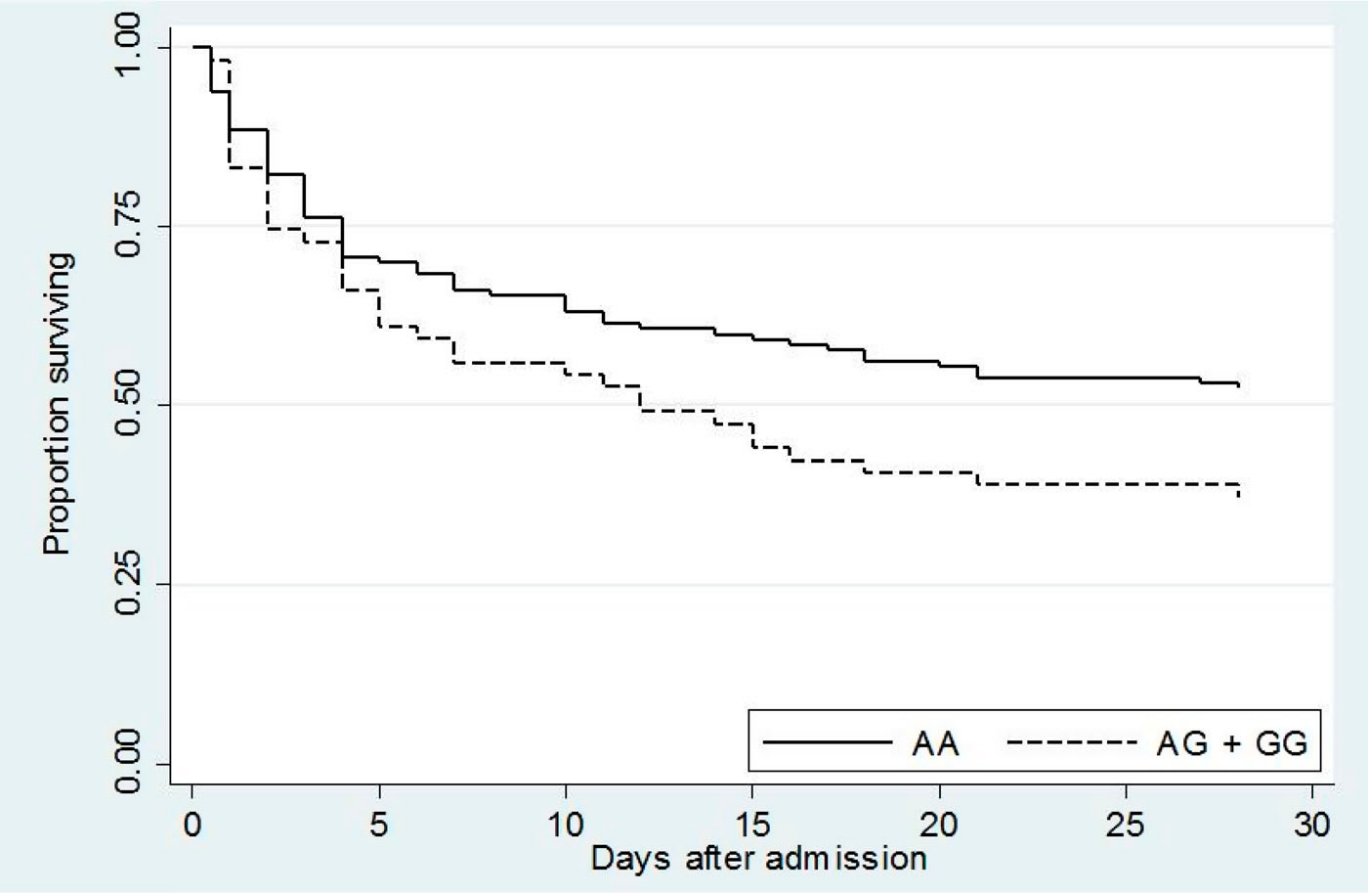
Kaplan–Meier survival curve of validation set subjects demonstrated a trend toward worse survival in carriers of rs76600635 (AG + GG). *P* = 0.07.

We also assessed the association of rs76600635 with survival in the validation set by Kaplan–Meier curve (Figure 2). The risk of death for subjects carrying the AG or GG genotype at this locus trended higher than for those who were not rs76600635 carriers (*P* = 0.07).

**Figure 3.**
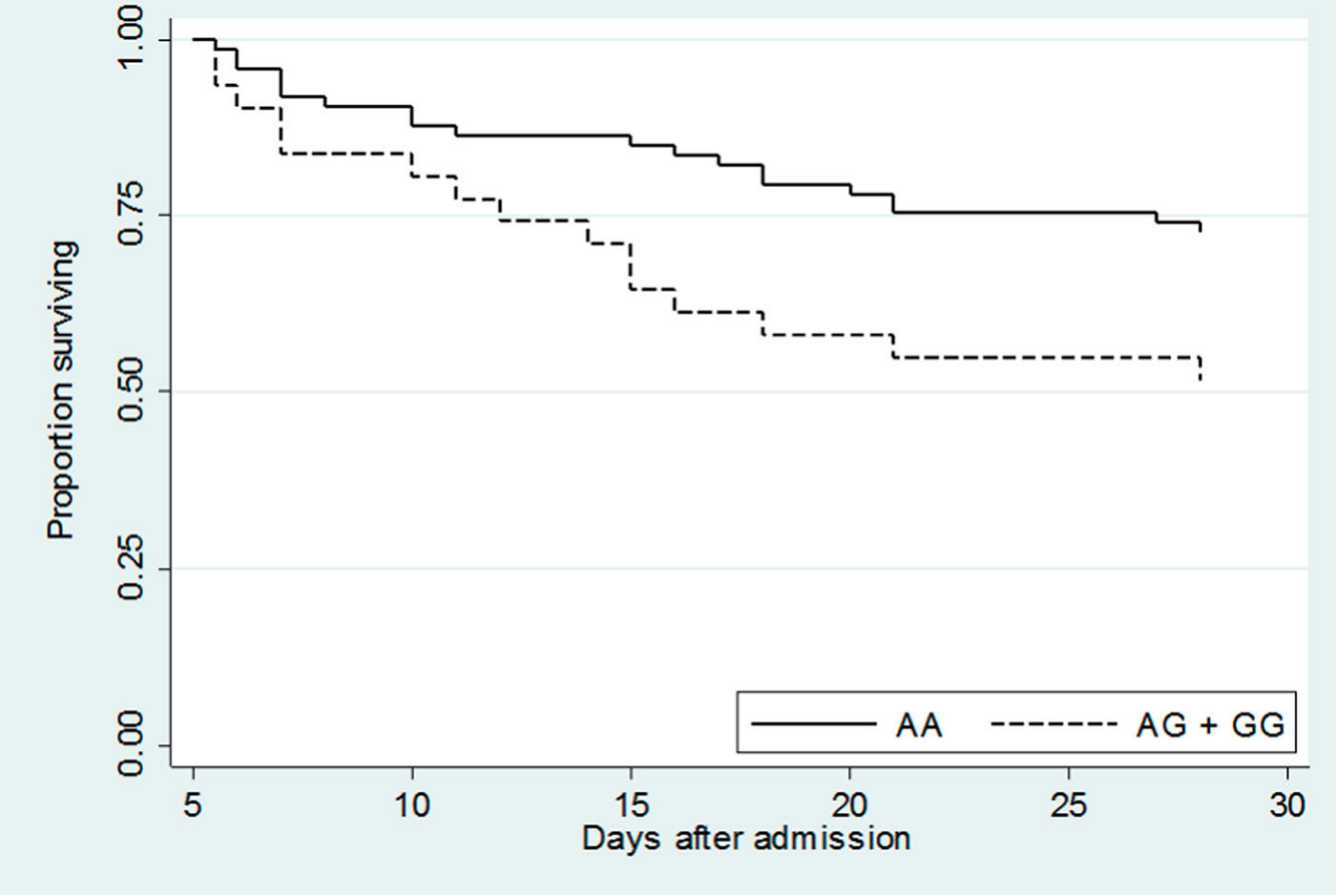
Kaplan–Meier survival curve of validation set subjects who were alive and hospitalized 5 days following admission demonstrated significantly lower survival in rs76600635 carriers (AG + GG) compared to non-carriers (AA). *P* = 0.03.

Due to differences in study design, subject recruitment into the validation set was typically performed earlier in the hospital stay than in the cohort used for the discovery or replication sets. This probably contributed to the high mortality rate (52%) observed in the validation set. To account for these differences in study design and thereby increase the comparability of our study cohorts, we repeated the analysis of rs76600635 restricted to those subjects alive and still hospitalized five days following admission. Although the time from admission to enrolment was not recorded for the cohort from which discovery or replication sets were derived, five days is the median duration of time from admission to enrolment following culture positivity in another study of patients with melioidosis at this hospital [17]. Using this approach, in 104 patients hospitalized five days after admission with melioidosis, 28-day mortality was 34% (35/104). Carriage of the rs76600635 variant was associated with death in both an unadjusted model [OR 2.48, 95% CI 1.03–5.96, *P* = 0.04] and an adjusted model [adjusted OR 3.88, 95% CI 1.43–10.56, *P* = 0.01] (Table 5). Furthermore, in the survival analysis of this restricted set of patients, rs76600635 carriers had significantly worse survival compared to non-carriers (*P* = 0.03) (Figure 3).

**Table 5.** Association of *TLR1* variant rs76600635 with 28-day mortality in subjects who were alive and hospitalized 5 days following admission in validation set subjects.

Variant	Genotype	28-day mortality		Unadjusted			Adjusted^a^		
		Survivor (*n* = 69)	Non-survivor (*n* = 35)	OR	95% CI	*P* value	OR	95% CI	*P* value
rs76600635	AA	53 (77%)	20 (57%)						
	AG	15 (22%)	14 (40%)	2.48	1.03–5.96	0.04	3.88	1.43–10.56	0.01
	GG	1 (1%)	1 (3%)						

^a^For adjusted model, values were determined using logistic regression analyses adjusted for age, sex, prior history of diabetes mellitus and chronic renal insufficiency.

## Discussion

Melioidosis is an important cause of sepsis in certain tropical regions and particularly in northeast Thailand [2]. However, the etiology of the varying phenotypic responses to melioidosis is unknown, even when accounting for co-morbid risk factors [6,31]. A paucity of data exists regarding the role of genetic variation in these diverse presentation types. Prior studies evaluating *TLR1* variants associated with sepsis outcomes in North American populations have not extended to Thai populations with melioidosis [25]. However, our results now implicate genetic variation in *TLR1* in the pathway leading to mortality in melioidosis.

rs76600635 is a missense variant in the *TLR1* region of chromosome 4 resulting in an amino acid change from a serine to a proline. The overall MAF of the variant is 0.10 in five East Asian populations of the 1000 Genomes Project in Ensembl. The MAF is 0.09–0.13 in three Chinese populations, 0.05 in a single Japanese population, and 0.13 in a single Vietnamese population [32]. While we found similar frequencies across our cohorts, the frequency of this variant in the broader population of Thailand is unknown. Notably, this variant has not been reported in European, African or Native American populations [30,33]. Therefore, while rare in populations across the global population, this variant may be relatively common in East and Southeast Asia.

While rs76600635 was recently associated with thrombocytopenia in Western Chinese patients undergoing treatment for tuberculosis, to the best of our knowledge no other clinical associations have been reported [34]. In a prior study, when a TLR1 construct with the rs76600635 variant was transfected into HEK cells and stimulated with Pam_3_CSK_4_, a TLR1 agonist, no change in NF-κB activation was observed [35]. However, the exact conformational consequences of this missense variant on receptor proteins is unknown, with a predicted “moderate” effect by Ensembl VEP [29,36]. Due to the paucity of data regarding this *TLR1* variant, multiple explanations exist regarding a potential mechanistic effect. First, rs76600635 may impart a deleterious effect on the innate immune response to melioidosis, increasing the risk of death. Second, as rs76600635 is not associated with altered NF-KB-mediated responses, the effect of the variant may represent a compromise of adaptive immune responses in those who survive longer given that *TLR1* variants are linked to T regulatory and adaptive immune responses [37,38]. Third, rs76600635 may tag a functional variant. For example, analysis of the 1000 Genomes Project indicates that in East Asian populations, rs76600635 is in linkage disequilibrium (LD) with two other variants (*r*^2^ > 0.8). One of these LD variants, rs77697303, is in a non-coding region of *TLR1*. The other LD variant, rs72493538, is a synonymous variant in a coding region of *TLR1* with no reported clinical associations and of unknown functional consequence.

While the adjusted OR point estimate for the association of rs76600635 with death in the validation set (1.81) was similar to that measured in the replication set (1.71), the confidence interval crossed 1 and thus did not meet statistical significance. This may reflect a true negative association, inadequate power to detect a significant effect in the smaller validation set, or that the recruitment strategies for the discovery, replication and validation sets differed. Enrolment into the cohort from which the discovery and replication sets were derived tended to be earlier in the hospitalization than for the sepsis cohort from which the validation set was derived; therefore some subjects who died or were discharged early from the hospital may not have been captured in the discovery and replication sets. Furthermore, the sepsis cohort that furnished the validation set recruited subjects who were inpatients on a medical service whereas surgical patients were more commonly enrolled in the cohort furnishing the discovery/replication sets. These differences may explain why the mortality rates of the replication (19%) and validation sets (50%) differed substantially.

To address the differences in recruitment, we performed a secondary analysis on subjects in the validation set who were still hospitalized at 5 days, Analysed in this way, the mortality rates between the replication (19%) and the validation sets (31%) begin to converge and may represent a more accurate group for comparison. This secondary analysis demonstrated a significant association between rs76600635 carriers for both higher mortality and worse survival and suggests this variant may have stronger effects on late mortality in melioidosis. We speculate that a deleterious effect of rs76600635 may be overwhelmed by other biological processes in those patients who are profoundly septic and in moribund condition.

Our discovery set identified a *TLR1* variant, rs4833095, which has been previously evaluated as a candidate variant in this cohort by our group [25]. The present analysis demonstrated a similar MAF to what we previously reported as well as the lack of an association with mortality. The rs4833095 allele is associated with mortality in Gram-positive, trauma-related sepsis patients in North America [39]. However, haplotypes tagged by rs4833095 are markedly different in Asian compared to white North American and European populations [40].

This study has some potential limitations. Extensive LD exists with the *TLR6-TLR1-TLR10* loci on chromosome 4. While we determined the association of one variant within *TLR1* with melioidosis-related mortality, we cannot exclude the possibility that a causative variant in LD with our subset of variants exists or that our observations are explained by unmeasured confounding such as population stratification. Furthermore, the three sets of melioidosis patients were all recruited at a single hospital and the results may not be generalizable to a broader population. Also, culture-positive patients with melioidosis may have died prior to enrolment or were enrolled with unaccounted for co-infections.

In summary, in three sets of Thai subjects with melioidosis, we identified a novel *TLR1* variant associated with poor outcomes. As the rs76600635 variant has not been previously associated with outcomes in sepsis, additional studies of this TLR1 variant and its potential mechanistic role in the host immune response to melioidosis and other causes of sepsis in East Asian subjects are required.

## Material and methods

### Study design

This study was separated into 3 phases: discovery, replication and validation. In the discovery phase, patients with bacteremic melioidosis and extreme clinical phenotypes had whole exome sequencing performed with *a priori* classification of *TLR1* as a gene of interest. Extreme phenotypes were defined as patients on the furthest spectrums of disease outcome: those with mild clinical disease who survived and those with severe clinical infection who died with five days of admission. In the replication phase, patients with culture-proven melioidosis were genotyped for *TLR1* variants identified during the discovery phase. In the validation phase, patients with evidence of sepsis and culture-proven melioidosis were genotyped for a single *TLR1* variant identified during the discovery and replication phases.

### Patient cohorts

#### Discovery and replication sets

These sets were derived from a cohort of patients aged 18 years or older admitted to Sunpasitthiprasong Hospital, Ubon Ratchathani, Thailand from 1999 to 2005 with melioidosis, as previously described [14]. In brief, hospitalized subjects with melioidosis were identified by active case finding by a study team. Melioidosis was defined as isolation of *B. pseudomallei* from any clinical sample (blood, sputum, endotracheal or tracheal aspirate, bronchoalveolar lavage, purulent fluid or urine) obtained by the study team or the hospital clinicians. Whole blood samples were obtained at the time of enrolment.

A nested case–control analysis was performed on 87 patients with blood culture-positive melioidosis who met criteria for extreme phenotypes of melioidosis. These bacteremic patients constituted the discovery set. Forty-two patients were classified as a severe phenotype of melioidosis, defined as organ failure and death within five days of admission. Forty-five patients were classified as a mild phenotype of melioidosis, defined as no evidence of organ failure and survival to discharge from hospital. Whole exome sequencing was performed on peripheral blood leukocyte DNA.

The replication set was comprised of 459 patients with melioidosis (culture positive from any sample). Genotyping was performed on peripheral blood leukocyte DNA.

#### Validation set

This set was derived from a cohort of patients aged 18 years or older admitted to Sunpasitthiprasong Hospital, Ubon Ratchathani, Thailand from 2013 to 2017 with suspected or documented infection and at least three sepsis diagnostic criteria, according to the 2012 Surviving Sepsis Campaign [41]. These patients were prospectively enrolled within 24 h of admission. Subsets of this sepsis cohort have been previously reported [42,43]. One hundred and ninety three sepsis patients met criteria for melioidosis based on a subsequently positive culture for *B. pseudomallei* obtained by the study team or hospital clinicians. Blood samples were obtained at the time of enrolment. Genotyping was successfully performed on peripheral blood leukocyte DNA from 189 patients.

### Whole exome sequencing

In the discovery set, DNA was extracted from peripheral blood using the QIAamp DNA Blood Midi Kit (Qiagen, Hilden, Germany). Whole exome sequencing of DNA was performed at the University of Washington Northwest Genomics Center. Exome capture was performed using Roche Nimblegen SeqCap EZ Exome V2.0 followed by sequencing with Illumina HiSeq, with base calling and assembly done with GATK software tools, version 3.1.

### Genotyping

In the replication set, DNA was extracted from blood using the QIAamp DNA Blood Midi Kit (Qiagen, Hilden, Germany) and *TLR1* variants were genotyped using the GoldenGate platform (Illumina, San Diego, CA, USA). In the validation set, DNA was extracted from blood using the Wizard Genomic DNA Purification Kit (Promega, Madison, WI, USA) and rs76600635 was genotyped with a TaqMan SNP genotyping assay (Applied Biosystems, Foster City, CA, USA) on a ViiA7 Real-Time PCR System (Applied Biosystems, Foster City, CA, USA).

### Statistical analysis

For the discovery set, *TLR1* variants identified in the mild and severe extreme phenotypes were analysed by performing tests of association between phenotypes and variants within the gene using the small-sample-adjusted sequence kernel association test (adj-SKAT-O), a variance components test [44,45].

In the replication and validation sets, deviation from Hardy–Weinberg equilibrium was calculated for each analysed variant. The crude association between genotype and mortality was performed using the chi-square test. Unadjusted and adjusted analyses, assuming dominant genetic models, were performed using logistic regression. Adjusted analyses included as covariates age, sex, prior history of diabetes mellitus and chronic kidney disease. Survival analyses were performed by generating Kaplan–Meier curves and comparisons were made using the logrank test.

Demographic analyses were performed using either the chi-square or Fisher's exact tests. Analyses were performed using Stata version 14.2 (College Station, TX, USA). Two-sided *P* values < 0.05 were considered significant.

### Ethics statement

These studies were approved by the Ethical Review Committee for Research in Human Subjects, Ministry of Public Health, Thailand; the Ethics Committee of the Faculty of Tropical Medicine, Mahidol University, Bangkok, Thailand; the Ethical Review Committee for Research in Human Subjects, Sunpasitthiprasong Hospital, Ubon Ratchathani, Thailand; the Oxford Tropical Medicine Ethics Committee, Oxford UK; and the University of Washington Human Subjects Division Institutional Review Board. Written informed consent for enrolment in the clinical studies was obtained from subjects or their representatives at the time of enrolment.
